# Supplementary material for: Spatial patterns associated with the distribution of immature stages of Aedes aegypti in three dengue high-risk municipalities of Southwestern Colombia
Source: GigaByte. 2023 Oct 27;2023:gigabyte95. doi: 10.46471/gigabyte.95 (PMC10620433; doi:10.46471/gigabyte.95)
Supplement: Translation

# Translation: Patrones espaciales asociados a la distribución de estadios inmaduros de *Aedes aegypti* en tres municipios de alto riesgo para el departamento del Cauca, Colombia

**Authors:** Cristina Sánchez Gutierrez, Erika Santamaría, Carlos Andrés Morales, María Camila Lesmes, Horacio Cadena, Alvaro Avila-Diaz, Patricia Fuya, Catalina Marceló-Díaz

**Affiliations:** ^1^ Grupo de Entomología, Instituto Nacional de Salud, Bogotá, Colombia; ^2^ Secretaría de Salud Departamental del Cauca, Popayán, Colombia; ^3^ Universidad de Ciencias Aplicadas y Ambientales, Bogotá, Colombia; ^4^ Programa de Estudio y Control de Enfermedades Tropicales PECET, Medellín, Colombia; ^5^ Research Group “Interactions Climate-Ecosystems (ICE)”, Earth System Science Program, Faculty of Natural Sciences, Universidad del Rosario, Bogotá, Colombia

## Abstract

Los mosquitos *Aedes aegypti* son el principal vector de las arbovirosis humanas en zonas tropicales y subtropicales. Su adaptación a entornos urbanos y rurales genera infestaciones en el intradomicilio de las viviendas. De aquí que, la vigilancia entomológica en asociación con el análisis espacial y el análisis espacio-temporal sean un enfoque innovador para el control de vectores y la gestión del dengue. El objetivo principal de la investigación fue realizar una comparación de la vigilancia entomológica, mediante el uso de índices cuantitativos de pupas y de adultos en tres municipios de alto riesgo de dengue en el departamento del Cauca, con el fin de examinar cómo influye la productividad de pupas, entre otros índices entomológicos, en la densidad de mosquitos adultos y sus patrones espaciales y temporales. Como resultado aportamos datos novedosos sobre la distribución geográfica de 3.806 estadios inmaduros de *Ae. aegypti*, sus índices entomológicos y su caracterización espacial. Los resultados sugieren que, para las especies de *Ae. aegypti*, la productividad de pupas genera altas densidades de adultos en las viviendas vecinas, evidenciando un comportamiento estacional. Nuestro conjunto de datos es esencial, ya que proporciona una estrategia innovadora para entender las enfermedades transmitidas por vectores utilizando patrones espaciales de vectores. También delinea la asociación entre estos patrones espaciales de vectores, indicadores entomológicos y criaderos ubicados en barrios de alto riesgo.

## Descripción de los datos

### Introducción

El mosquito *Aedes aegypti* es el principal vector de las arbovirosis humanas, tales como: fiebre amarilla, dengue, chikungunya y Zika, las cuales son prevalente en áreas tropicales y subtropicales [1.]. Su adaptación a condiciones antrópicas locales, ambientes urbanos, condiciones óptimas de temperatura, precipitación, humedad relativa entre otros factores ambientales y socioeconómicos le han conferido la capacidad de establecer sus poblaciones reproductivas entre las poblaciones urbanas y rurales [2.–4.]. Para el caso de Colombia, *Ae. aegypti* es el principal vector del virus dengue reportándose en altitudes entre el nivel del mar hasta los 2302 m. Por lo tanto, la presencia en un amplio rango altitudinal y la abundancia de este vector son una amenaza a la salud pública del país. [5., 6.].

El estrecho contacto con los seres humanos ha permitido que la hembra de *Ae. aegypti* de hábitos hematófagos se alimente de sangre humana o de animales domésticos; causando infestaciones en diferentes tipos de contenedores en los domicilios y sus alrededores. En ese sentido, la presencia de contenedores artificiales y naturales como tanques, barriles, botellas, neumáticos, plantas acuáticas (bromelias y agua retenida en la parte axilar de las plantas), agua estancada, bebederos de mascotas, entre otros, se convierten en criaderos potenciales de estados inmaduros del mosquito *Ae. aegypti* [7., 8.]. La vigilancia de este tipo de contenedores contribuye a reducir la proliferación de pupas y por ende densidades del mosquito, su muestreo se convierte en una herramienta indispensable para diseñar enfoques de vigilancia entomológica del vector, pertinentes a cada situación [9.].

De acuerdo con la metodología para la búsqueda activa de potenciales criaderos establecida para Colombia [7., 10.], la inspección se determina con indicadores entomológicos que permiten dar a conocer el índice de pupas por persona, productividad de pupas y la presencia de estadios inmaduros en las viviendas [7., 10.]. Sin embargo, esto no se lleva a cabo en todas las entidades territoriales. La importancia de emplear este tipo de indicadores entomológicos es que proporcionan una aproximación representativa de las poblaciones locales adultas del mosquito requiriendo un tamaño muestral de inspección de casas relativamente pequeño [9., 11.]. Por otra parte, el índice de pupas por persona posibilita calcular un umbral mínimo de infestación por pupas de *Ae. aegypti* para el riesgo de transmisión del virus dengue (DENV). Específicamente si este índice oscila entre 0,5 a 1,5 para temperaturas de 28 °C y seroprevalencias entre 0% a 67% [12.], permite esclarecer zonas con mayor riesgo.

La vigilancia del mosquito *Ae. aegypti* se ha basado principalmente en la obtención de los índices de larvas cualitativos, los cuales guardan poca relación con el número de hembras del mosquito. Por otra parte, se ha reportado que las poblaciones de *Ae. aegypti* son espacialmente heterogéneas en algunas zonas [11.]. Así pues, el uso del análisis espacio-temporal para predecir la aparición de puntos calientes de mosquitos *Ae. aegypti*, es decir, grupos de casas con elevados incrementos en la productividad de pupas, alta densidad del vector y potencial transmisión del DENV, es una de las estrategias innovadoras que pueden utilizarse para la prevención, control del vector y la gestión integrada del dengue en municipios de alto riesgo. Esto se debe a que este método permite detectar patrones de distribución geográfica del vector [11., 13.]. Por tal motivo, es de suma importancia considerar este tipo de índices de infestación debido que los estadios inmaduros, específicamente las pupas representan el estadio anterior próximo al adulto con muy baja mortalidad. De esta manera aumenta la correlación entre la densidad de *Ae. aegypti* y el virus del dengue [7., 14.].

Es así que, en este estudio, se comparó la vigilancia entomológica, mediante el uso de índices cuantitativos de pupas y de adultos en tres municipios de alto riesgo de dengue: Patía (El Bordo), Miranda y Piamonte en el departamento del Cauca. Principalmente con el fin de examinar cómo influye la productividad de pupas, entre otros índices entomológicos, en la densidad de mosquitos adultos y sus patrones espaciales y temporales. Para ello, se llevaron a cabo análisis de regresión espacial y de autocorrelación espacial bivariada, que permitieron modelar y explorar las relaciones espaciales, además de captar la relación entre los valores de dos variables, aun cuando estas no coincidían en el mismo espacio.

## Contexto

Se documentan 160 registros biológicos (3.806 individuos) en estadio inmaduro (pupa) de la especie *Ae. aegypti* provenientes de las viviendas de los municipios de Patía (El Bordo), Miranda y Piamonte; ubicados en el departamento del Cauca, al suroccidente de Colombia. Estos municipios forman parte de un proyecto de investigación en curso titulado “Estratificación espacial del dengue basado en la identificación de factores del riesgo: un ensayo piloto en el departamento del Cauca”, liderado por el Grupo de Entomología del Instituto Nacional de Salud, la Secretaría de Salud del Cauca y la Universidad de Ciencias Aplicadas y Ambientales (UDCA). El cual busca entre otros, evaluar la fuerza de asociación de las variables entomológicas en la explicación del patrón espacial observado en la distribución del dengue.

Los datos fueron recopilados entre los años 2021 y 2022 por un equipo multidisciplinar de técnicos de sanidad ambiental, ingenieros geógrafos y ambientales, y profesionales con amplia experiencia en entomología médica. En este conjunto de datos se diferenciaron tres periodos de muestreo para cada municipio, para Patía: marzo y octubre del 2021 y marzo y abril del 2022; para Miranda: abril y noviembre del 2021 y mayo del 2022; y para Piamonte julio del año 2021 y febrero y julio del 2022. Un cuarto período de muestreo se llevó a cabo en agosto y octubre del 2022 en los municipios de Patía y Miranda respectivamente, con el fin de incrementar el tamaño de muestra para llevar a cabo serotipificación de los ejemplares.

Los estadios inmaduros (pupas) fueron recolectados de recipientes menores y mayores de 20 litros incluyendo, tanques, barriles, neumáticos, entre otros, provenientes del intradomicilio de las viviendas de los tres municipios. Los municipios fueron seleccionados como área de estudio por su comportamiento endemo-epidémico para la transmisión del virus dengue (DENV), caracterizado por presentar endemias focales, escenarios de transmisión heterogéneos, patrones temporales y cíclicos en poblaciones de riesgo.

El conjunto de datos de estadios inmaduros está en formato Darwin Core, con 73 términos disponibles. Todos los campos obligatorios están presentes y han sido sometidos a revisión en el IPT (Integrated Publishing Toolkit) del SiB-Colombia. Los campos de metadatos también están disponibles a través de las páginas disponibles en línea [15.]. También hacen parte de este conjunto de datos 3.480 individuos adultos de la familia Culicidae [16.], de los cuales alrededor del 69% de los especímenes fueron colectados en el año 2021, mientras que 1.078 individuos fueron nuevos registros recolectados en el año 2022.

Estos registros biológicos son fundamentales para la comunidad científica ya que proporcionan las coordenadas geográficas, así como índices entomológicos necesarios para la vigilancia en salud pública que se lleva a cabo por parte de las entidades de salud. En particular, estas medidas son esenciales para construir estrategias que permitan prevenir y controlar el dengue en viviendas y barrios con altos índices de infestación del vector.

## Métodos

### Muestreo

Los especímenes de los estados inmaduros de *Ae. aegypti* fueron recolectados en viviendas ubicadas en los municipios de Patía (El Bordo), Miranda y Piamonte en el departamento del Cauca, Colombia (Figure 1). Estos municipios fueron seleccionados por considerarse endemo-epidémicos con alto riesgo de transmisión del dengue, después de realizar un análisis espacio temporal [16.]. El tamaño de muestra fue delimitado a una escala espacial fina por manzanas mediante análisis de densidad de Kernel, georreferenciando los casos de dengue reportados en el periodo de 2015 a 2019 en áreas urbanas de cada municipio, adicionalmente el tamaño de la muestra se calculó a partir de la prevalencia estimada de dengue en el municipio (10,5%) [17.] con un nivel de confianza del 99% mediante el software Epi Info™, utilizando el tamaño de población estimado y los conglomerados obtenidos en el análisis Kernel.

**Figura 1.** Mapa interactivo de las ocurrencias (especímenes) georreferenciadas depositadas en GBIF [18.].https://www.gbif.org/dataset/a51b1fb9-6e67-42b9-879b-5f21e4d85642.

 En total, se visitaron 1.919 viviendas en 26 barrios durante los meses de marzo, abril, julio y octubre del año 2021, y febrero, marzo, mayo, julio, agosto y octubre de 2022. En el municipio de Patía se visitaron 11 barrios (*n* = 580 viviendas), en Miranda 12 barrios (*n* = 854 viviendas) y en Piamonte cuatro barrios (*n* = 485 viviendas).

### Recolección de especies

Se ejecutó una inspección diurna de las viviendas entre las 8:00 y las 17:00 horas, posteriormente se siguieron los parámetros estipulados de la guía Gestión para la vigilancia entomológica y control de la transmisión de dengue [7., 10.]. Se seleccionaron los depósitos de aguas artificiales que se encontraron en el intradomicilio. Se inspeccionan todos los criaderos sin importar su tamaño, es decir, contenedores menores a 20 L como botellas, floreros, latas, llantas y plásticos pequeños, desde donde fueron recolectadas las pupas con una pipeta Pasteur para su posterior conteo. Para el caso de contenedores mayores a 20 L se contó el número de pupas recolectadas con una jama y se valoró el volumen del depósito, y nivel del agua existente para calcular el factor de calibración [7.] y obtener el número estimado de pupas o productividad de pupas.

Se realizaron inspecciones entomológicas en busca de mosquitos adultos durante meses específicos del año 2021 (febrero, marzo, abril, julio, octubre y noviembre) y 2022 (febrero, marzo, mayo y julio). Estas inspecciones se realizaron entre las 8:00 y las 17:00 horas, con una duración media de 10 minutos por vivienda. En cada hogar se buscaron mosquitos adultos en la sala, comedor, baños, cocinas, patio de lavado y otras áreas y se capturaron utilizando un aspirador Prokopack. Estos procedimientos se llevaron a cabo haciendo énfasis en la búsqueda en áreas sombreadas o lugares cercanos a recipientes con agua durante el recorrido.

### Clasificación de especies y caracterización espacial

Posterior a la captura de los estadios inmaduros y adultos, se identificó y se clasificó taxonómicamente la especie *Ae. aegypti* por expertos entomólogos con el apoyo de las claves taxonómicas desarrolladas por Forattini [19.] (1995) y Harrison *et al.*, [20.] (2016) para diferenciarlas de estadios inmaduros pertenecientes a otras especies. Continuando con el protocolo de campo, se identificaron 3.806 pupas de *Ae. aegypti*, de ellas, 395 fueron conservadas en viales de 0,2 mL con RNAlater^©^ para su procesamiento con técnicas de biología molecular en el grupo de Entomología y el grupo de Genómica de Microorganismos Emergentes del Instituto Nacional de Salud (Bogotá, Colombia) [21.].

La información entomológica obtenida fue registrada empleando la aplicación de ArcGIS^®^ Survey 123 [22.], la cual permitió obtener la ubicación geográfica de cada espécimen recolectado. Se asoció un código a cada vial con la información sociodemográfica de la encuesta y a partir del número total de pupas registradas en cada encuesta, se determinaron los índices entomológicos de productividad de pupas, productividad de hembras, índice de pupas por persona, índice pupal de Breteau [7., 10.] y proporción de individuos F:M de pupas *Aedes* sp. (Tabla 1).

**Tabla 1:** Indicadores entomológicos para la vigilancia de *Ae. aegypti* [7., 9., 10.].

Indicador	Cálculo	Interpretación
Productividad de pupas	(# pupas recolectadas * Factor de calibración)	Calcula el número estimado de pupas por cada depósito. Depósitos menores a 20 L se cuenta únicamente el número de pupas. Depósitos mayores a 20 L se multiplica por factor de calibración (f.c.) proporcionada por el nivel del agua. **Niveles de agua:** <1/3 f.c. no aplica; 1/3 f.c.: 2.6; 2/3 f.c.: 3.0; 3/3 f.c.: 3.5.
Productividad de hembras	(La razón hembra:macho es de 1:1)	Establece el número estimado de hembras producidas por el criadero.
Índice de pupas por persona	(Productividad de pupas/# habitantes)	Genera una estimación del número de pupas por persona en la vivienda inspeccionada
Índice pupal Breteau	(Número de depósitos positivos con pupas * 100/Número de viviendas inspeccionadas)	Suministra el número de depósitos con pupas por cada 100 casas. Determina una relación entre los depósitos positivos y las viviendas inspeccionadas.

La información entomológica en conjunto con la información de las manzanas geográficas de los municipios se empleó para realizar análisis de diagramas de dispersión (*R* ^2^, pendiente *b*, valor *p*), así como matrices de diagramas de dispersión para evaluar las relaciones entre las variables entomológicas: número de mosquitos adultos de *Ae. aegypti* [18.], número total de pupas, productividad de pupas, productividad de hembras, número de pupas por persona con respecto a la frecuencia de contenedores de tipo tanques bajos, contenedores diversos menores a 20 L y barriles.

A su vez, se realizó un análisis espacial mediante el Índice de Moran bivariante global (*p* ≤ 0,05) para detectar la distribución de las variables relacionadas con agrupaciones espaciales. El Índice de Moran se encuentra en un rango de −1 a 1, en donde −1 indica patrones de agrupación dispersos, 0 indica aleatoriedad y 1 asociación perfecta. Posteriormente, se ejecutó el análisis para el Índice de Moran Local que distribuyó los agrupamientos significativos (*p* = 0,05) de las manzanas de las viviendas con cuatro tipos de asociación espacial local (I) alto (*x*)-alto (*y*) indica zonas con valores elevados de la variable rodeados de valores superiores a la media de la variable a analizar, (II) bajo (*x*)-alto(*y*) indica zonas con valores bajos de la variable rodeada de zonas vecinas con valores por encima de la media de la variable, (III) bajo (*x*)-bajo (*y*) indica zonas con bajos valores de la variable rodeados de zonas con valores por debajo de la media de la variable y (IV) alto(*x*)-bajo (*y*) zonas con valores elevados de la variable rodeados de zonas por debajo de la media de la variable a analizar [13.].

Los anteriores análisis fueron realizados en dos épocas de muestreo en cada municipio, es decir, para el municipio de Patía (El Bordo) las muestras recolectadas en los meses marzo y octubre del año 2021, para Miranda las muestras correspondientes de abril y noviembre del año 2021; y para Piamonte las muestras de julio del año 2021 y febrero del año 2022 debido que entre esos meses se evidenció temporalidad en el patrón de precipitación de lluvias. Finalmente, los análisis se efectuaron en el programa GeoDa v1.20 [23.] y la visualización de los mapas en el software ArcGIS^®^ 10.8 (SCR_011081).

## Validación de datos y control de calidad

Se identificaron 3.480 especímenes adultos en 1.200 viviendas, de los cuales 1.459 correspondían a hembras y 2.021 a machos. En el año 2021 se documentaron 2.402 registros, mientras que en el año 2022 se añadieron 1.078 nuevos registros. Con respecto a la distribución por municipios, Miranda reportó 500 individuos (14,36%), Patía registró 1.305 individuos (37,5%), y Piamonte presentó los registros más altos con 1.675 (48,13%) individuos adultos. Cabe destacar que se encontraron 71 individuos en una sola vivienda perteneciente al municipio de Patía y otros 125 individuos en una vivienda diferente del municipio de Piamonte.

Adicionalmente, se registraron un total de 3.806 especímenes en estado inmaduro de *Ae. aegypti*, distribuidos en 160 registros (casas positivas del total de inspeccionadas). Para el municipio de Patía (El Bordo) se hallaron 1.493 individuos en 67 viviendas positivas, para Miranda 1.173 individuos en 58 viviendas y para Piamonte se encontraron 1.140 individuos en 35 viviendas. Los barrios con mayor cantidad de especímenes fueron Villa Los Prados (*n* = 519) y La Paz (*n* = 359) en Piamonte, seguido del barrio San Antonio (*n* = 391) en Miranda y los barrios Libertador (*n* = 441) y Olaya Herrera (*n* = 438) en el municipio de Patía. Cabe resaltar que, se encontraron 70 casas inspeccionadas con más de 15 pupas de *Ae. aegypti* al interior de la vivienda. El mayor número de especímenes detectados pertenecía a una casa del municipio de Piamonte, con 280 pupas (Tabla 2).

**Tabla 2:** Medidas entomológicas descriptivas por municipio de muestreo. El total de viviendas inspeccionadas positivas, se muestra como un total y como porcentaje para cada municipio [24., 25.].

Medida entomológica	Municipio			Total
	Patía	Miranda	Piamonte	
Número de viviendas inspeccionadas	580	854	485	1919
Número de habitantes en las viviendas inspeccionadas	2098	3735	1880	7713
# casas positivas con pupas de *Aedes* sp. (%)	67 (11,55%)	58 (6,79%)	35 (7,22%)	121
Número de contenedores inspeccionados	1142	1067	984	3193
# de contenedores positivos con pupas de *Aedes* sp. (%)	50 (4,38%)	43 (4,03%)	36 (3,66%)	129
# total de pupas *Aedes* sp.	425	530	389	1344
Productividad de pupas *Aedes* sp.	1493	1173	1140	3806
Relación hembra:macho de pupas de *Aedes* sp. F:M [24., 25.]	1:1	1:1	1:1	1:1
Número de pupas por persona	0,71	0,31	0,61	1,63
Índice de Bretau	8,62	5,04	7,42	21,08

Para la época lluviosa del muestreo, en el análisis de regresión espacial, se encontró una correlación positiva para los municipios. Específicamente con un coeficiente de determinación superior al 50% en los municipios de Patía (*R* = 0,7292; *R* ^2^ = 0,53) y Miranda (*R* = 0,7486; *R* ^2^ = 0,56) e inferior en Piamonte (*R* = 0,4252; *R* ^2^ = 0,18) entre la captura de pupas y adultos, lo que sugiere el uso del índice de adultos para la vigilancia entomológica. Además, se observó una autocorrelación espacial entre la presencia de viviendas con pupas positivas y una mayor densidad de adultos en las manzanas vecinas.

Para el municipio de Patía, la variable productividad de pupas (hembra) explicó el número de adultos de *Ae. aegypti* con un ajuste del modelo del 53,2% en la época de mayor precipitación y con un ajuste de 8,4% en la época de menor precipitación, presentando en ambos casos autocorrelación positiva entre las variables (Tabla 3). Lo cual permitió localizar los conglomerados en los que la productividad de pupas genera alta densidad de mosquitos a nivel de manzana (Figura 2).

**Figura 2.** Índice de Moran local bivariante para las variables productividad de pupas (*x*) y número de mosquitos adultos (*y*), en los municipios de Patía, Miranda y Piamonte (2021–2022).

 Variables como la frecuencia de floreros positivos cuando las precipitaciones alcanzaron sólo 1 mm (octubre 2021), explicaron la densidad de mosquitos adultos sólo en un 15%; mientras que en el periodo de mayor precipitación (211 mm, marzo 2021), el contenedor tanques bajos explicó el 37% de la densidad de mosquitos adultos. Esta comparación resulta de interés teniendo en cuenta que el clima del municipio de Patía presenta un comportamiento bimodal con picos en los meses de abril y noviembre [26.]. Nuestro análisis local (LISA) observó agrupaciones espaciales heterogéneas alto-alto y bajo-bajo para cada temporada, principalmente en el barrio Olaya Herrera (marzo y octubre de 2021).

Con respecto al municipio de Miranda, se evidenció un ajuste del modelo *R* ^2^ del 56% y autocorrelación espacial positiva en la época de menor precipitación, aunque con alto porcentaje de humedad relativa, entre las variables productividad de pupas hembra y el número de mosquitos adultos. Se identificó también la presencia de autocorrelación positiva para las variables frecuencia de tanques bajos positivos y productividad de pupas. No obstante, para la época de mayor precipitación y menor humedad relativa se encontró autocorrelación espacial negativa en las variables evaluadas, es decir, con patrones de agrupación cercanos a la aleatoriedad (Tabla 3).

**Tabla 3:** Resultados del análisis de regresión y autocorrelación espacial bivariada para las variables entomológicas del mosquito *Ae. aegypti* en Patía, Miranda y Piamonte, teniendo en cuenta la estacionalidad del muestreo.

Municipio	Estacionalidad	Variables (*X*, *Y*)	Parámetros			I Moran (IM)
			*R* ^2^	Pendiente *b*	*p* valor	
Patía (El Bordo)	Época lluviosa mayor precipitación Marzo 2021	% tanques bajos - PPP Productividad de pupas ♀ - PPP % tanques bajos - No. mosquitos adultos % tanques bajos - Productividad de pupas ♀ Productividad pupas ♀ - No. mosquitos adultos	0,232 0,327 0,126 0,374 0,532	0,082 0,313 0,053 0,191 0,530	0,000* 0,000* 0,006* 0,000* 0,000*	**0,264** **0,325** **0,132** **0,109** **0,108**
	Época lluviosa menor precipitación Octubre 2021	% tanques bajos - PPP Productividad de pupas ♀ - PPP % floreros - PPP % floreros - Productividad de pupas ♀ Productividad pupas ♀ - No. mosquitos adultos	0,560 0,680 0,262 0,150 0,084	0,106 0,262 0,587 0,701 0,145	0,000* 0,000* 0,000* 0,004* 0.035*	**−0,048** **0,034** **0,033** **0,115** **0,196**
Miranda	Época lluviosa menor precipitación Abril 2021	Productividad pupas ♀ - No. mosquitos adultos % tanques bajos - No. mosquitos adultos Productividad de pupas - No. mosquitos adultos (♀)	0,560 0,116 0,437	0,114 0,07 0,028	0,000* 0,001* 0,000*	**0,086** **0,051** **0,043**
	Época lluviosa mayor precipitación Noviembre 2021	Productividad pupas ♀ - No. mosquitos adultos % tanques bajos - No. mosquitos adultos Productividad de pupas - No. mosquitos adultos (♀)	0,043 0,000 0,045	0,366 −0,003 0,145	0,064 0,965 0.058*	**−0,035** **−0,029** **−0,047**
Piamonte	Época lluviosa mayor precipitación Julio 2021	Productividad pupas ♀ - No. mosquitos adultos % contenedores diversos < 20 L - PPP % barriles - Productividad de pupas ♀	0,181 0,001 0,054	0,037 −0,063 1,026	0,004* 0,809 0,123	**0,058** **0,054** **0,043**
	Época Seca bajas precipitaciones Febrero 2022	Productividad pupas ♀ - No. mosquitos adultos % contenedores diversos < 20 L - PPP % barriles - Productividad de pupas ♀ % baldes - Productividad de pupas ♀	0,197 0,006 0,107 0,377	0,085 0,004 0,247 1,357	0,005* 0,643 0,042* 0,000*	**0,044** **0,124** **0,112** **−0,026**

PPP: No. de pupas por persona. * *p* ≤ 0,05.

En el caso de Piamonte para la época lluviosa, la variable productividad de pupas hembra explicaron el ajuste del modelo (*R* ^2^) en un 18% con autocorrelación espacial bivariada positiva (Índice de Moran IM = 0,058). En relación con los contenedores diversos menores a 20 L, barriles y tanques bajos presentaron autocorrelación positiva no significativa referente a los indicadores entomológicos y número de adultos, resultados que contrastan con la correlación entre el porcentaje de baldes positivos y la productividad de pupas (*R* ^2^ = 37,7%) en la época seca del muestreo.

Los resultados sugieren que para la especie *Ae. aegypti*, la productividad de pupas genera altas densidades de adultos en las viviendas vecinas, lo que permite identificar con el indicador local de asociación espacial (LISA) las manzanas de los barrios donde esta tendencia ocurre, evidenciando un comportamiento estacional. Mientras que la correlación espacial vinculó la captura de pupas y adultos en el mismo espacio geográfico, la autocorrelación bivariada (IM) relacionó estas mismas variables, pero sin que coincidan necesariamente en la misma vivienda. Asimismo, entre municipios se observó una mayor autocorrelación espacial positiva entre pupas y adultos en Patía, especialmente cuando descienden las precipitaciones previo al inicio de la temporada de lluvia. Nuestros resultados son similares a otros estudios espacio-temporales relacionados con la densidad de mosquitos [13., 27.] mostrando patrones heterogéneos de ocurrencia para cada territorio, con comportamientos estacionales que registran mayores tasas de infestación en temporadas de mayor pluviosidad.

## Potencial de re-uso

La base de datos y el mapa de distribución del vector proporcionan importantes recursos para el entendimiento de los patrones espaciales del vector y su relación con indicadores entomológicos y con criaderos, que podrían aumentar la transmisión del virus dengue en los municipios estudiados.

Para mejorar la accesibilidad de estos datos, la base de datos ha sido incluida en el GBIF. Esta información será útil para realizar aproximaciones representativas de la densidad del mosquito, mapear zonas con elevados incrementos en la productividad de pupas, y relacionar otros determinantes ambientales, entomológicos o sociodemográficos que suministren información vital para generar estrategias innovadoras en prevención, control del vector y la gestión del dengue. Sugerimos a otros investigadores que hagan disponibles sus datos de manera similar.

## Data Availability

El conjunto de datos en los que se basa este artículo están disponibles en el repositorio GBIF [18.], así como en GigaDB [28.].

## Notas del editor

Este documento forma parte de una serie de artículos de divulgación de datos en colaboración con GBIF y con el apoyo de TDR, el Programa Especial de Investigaciones y Enseñanzas sobre Enfermedades Tropicales, acogido en la Organización Mundial de la Salud [29.].

## Abreviaciones

DENV, virus del dengue; IPT, Integrated Publishing Toolkit; LISA, indicador local de asociación espacial; IM, Índice de Moran; TWS, tanques para almacenamiento de agua; UDCA, Universidad de Ciencias Aplicadas y Ambientales.

## Declaraciones

### Aprobación ética y consentimiento para participar

Este trabajo fue aprobado por el Comité de Ética y Metodologías de la Investigación (CEMIN) del Instituto Nacional de Salud en Bogotá, Colombia (Proyecto de Investigación CEMIN 13-2019).

### Autorización de publicación

No aplicable.

### Contribución del autor

CS: curación de datos, redacción (borrador original, edición), análisis de datos, software, investigación; ES: investigación, supervisión, administración del proyecto, datos de campo, redacción (revisión y edición); CAM, conceptualización, administración del proyecto, datos de campo, redacción (revisión y edición); MCL, datos de campo, curación de datos, visualización; HC, redacción (revisión y edición); AAD, redacción (revisión y edición); PF, redacción (revisión y edición, datos de campo; CMD, conceptualización, curación de datos, software, visualización, redacción (borrador original, edición), metodología, administración del proyecto, obtención de fondos, investigación. Todos los autores hicieron comentarios al manuscrito.

### Financiación

Este trabajo fue financiado por el Instituto Nacional de Salud, Bogotá, Colombia (proyecto de investigación CEMIN 13-2019), la Secretaría de Salud Departamental del Cauca, la Universidad de Ciencias Aplicadas y Ambientales UDCA y el Ministerio de Ciencia, Tecnología e Innovación de Colombia (proyecto de investigación 210484467217).

## References

[1] López-LatorreMA, NeiraM. Influencia del cambio climático en la biología de *Aedes aegypti* (Diptera: Culicidae) mosquito transmisor de arbovirosis humanas. Rev. Ecuat. Med. Cienc. Biol., 2016; 37(2): 11–21. ISSN: 2477-9148.

[2] TalbotB, SanderB, CevallosV Determinants of *Aedes* mosquito density as an indicator of arbovirus transmission risk in three sites affected by co-circulation of globally spreading arboviruses in Colombia, Ecuador and Argentina. Parasites Vectors, 2021; 14(1): 482. doi:10.1186/s13071-021-04984-z.34538276PMC8451087

[3] Vásquez-TrujilloA, Cardona-ArangoD, Segura-CardonaAM House-level risk factors for *Aedes aegypti* Infestation in the Urban Center of Castilla la Nueva, Meta State, Colombia. J. Trop. Med., 2021; 2021: 8483236. doi:10.1155/2021/8483236.34725551PMC8557085

[4] OvergaardHJ, OlanoVA, JaramilloJF A cross-sectional survey of *Aedes aegypti* immature abundance in urban and rural household containers in central Colombia. Parasites Vectors, 2017; 10(1): 356. doi:10.1186/s13071-017-2295-1.28750651PMC5530958

[5] Ruiz-LópezF, González-MazoA, Vélez-MiraA Presencia de *Aedes (Stegomyia) aegypti* (Linnaeus, 1762) y su infección natural con el virus del dengue en alturas no registradas para Colombia. Biomedica, 2016; 36(2): 303–308. doi:10.7705/biomedica.v36i2.3301.27622492

[6] NelsonMJ. *Aedes aegypti:* biologia y ecologia. Organización Panamericana de la Salud. 1986; https://iris.paho.org/handle/10665.2/28513. Accessed 18 February 2022.

[7] Romero-VivasCM, LlinásH, FalconarAK. Three calibration factors, applied to a rapid sweeping method, can accurately estimate *Aedes aegypti* (Diptera: Culicidae) pupal numbers in large water-storage containers at all temperatures at which dengue virus transmission occurs. J. Med. Entomol., 2007; 44(6): 930–937. doi:10.1603/0022-2585(2007)44[930:tcfata]2.0.co;2.18047190

[8] HaT-A, LeónTM, LalanguiK Household-level risk factors for *Aedes aegypti* pupal density in Guayaquil, Ecuador. Parasites Vectors, 2021; 14(1): 458. doi:10.1186/s13071-021-04913-0.34493321PMC8425057

[9] BarreraR. Recommendations for the surveillance of *Aedes aegypti* . Biomedica, 2016; 36(3): 454–462. doi:10.7705/biomedica.v36i3.2892.27869394PMC6537883

[10] Ministerio de la Protección Social República de Colombia. Instituto Nacional de Salud. Organización Panamericana de la Salud. Anexo 8. Procedimientos para la recolecta de larvas, pupas y mosquito adultos de *Aedes aegypti*. En: Gestión para la Vigilancia entomológica y control de la transmisión de dengue. Colombia. 2011.

[11] LaConG, MorrisonAC, AsteteH Shifting patterns of *Aedes aegypti* fine scale spatial clustering in Iquitos, Peru. PLoS Negl. Trop. Dis., 2014; 8(8): e3038. doi:10.1371/journal.pntd.0003038.25102062PMC4125221

[12] FocksDA, BrennerRJ, HayesJ Transmission thresholds for dengue in terms of *Aedes aegypti* pupae per person with discussion of their utility in source reduction efforts. Am. J. Trop. Med. Hyg., 2000; 62(1): 11–18.10761719

[13] CavalcanteACP, de OlindaRA, GomesA Spatial modelling of the infestation indices of *Aedes aegypti*: an innovative strategy for vector control actions in developing countries. Parasites Vectors, 2020; 13(1): 197. doi:10.1186/s13071-020-04070-w.32299496PMC7164210

[14] AlcaláLA, QuinteroJ, GonzálezC Productividad de *Aedes aegypti* (L.) (Diptera: Culicidae) en viviendas y espacios públicos en una ciudad endémica para dengue en Colombia. Biomedica, 2015; 35(2): 258–268. doi:10.7705/biomedica.v35i2.2567.26535548

[15] Sistema de Información sobre Biodiversidad de Colombia - SiB IPT. https://ipt.biodiversidad.co/sib/.

[16] Marceló-DíazC, MoralesCA, LesmesMC Arbovirus vectors in municipalities with a high risk of dengue in Cauca, Southwestern Colombia. GigaByte, 2022; 2022: gigabyte53. doi:10.46471/gigabyte.53.36824502PMC9930565

[17] PadillaJ, RojasD, Sáenz-GómezR. Dengue en Colombia: epidemiología de la reemergencia a la hiperendemia. Revista Salud Bosque, 2012; 5(1): 81–83. doi:10.18270/rsb.v5i1.186.

[18] Marceló-DíazC, MoralesCA, FuyaOP Registros de los dípteros causantes de la transmisión del agente etiológico del dengue en el departamento del Cauca, Colombia. Version 1.8. Instituto Nacional de Salud. 2023; Occurrence dataset 10.15472/dxbowv accessed via GBIF.org on 2023-04-19.

[19] ForattiniOP. Principais mosquitos de importância sanitária no Brasil. Cad. Saúde Pública, 1995; 11(1): 157–158. doi:10.1590/S0102-311X1995000100027.

[20] HarrisonBA, ByrdBD, SitherCB The Mosquitoes of the Mid-Atlantic Region: An Identification Guide. Cullowhee, North Carolina: Western Carolina University, 2016. ISBN: 9780692646359.

[21] Instituto Nacional de Salud Columbia Website. http://www.ins.gov.co.

[22] ArcGIS Survey123 Website. https://survey123.arcgis.com/.

[23] GeoDa Website. https://geodacenter.github.io/.

[24] FocksDA, AlexanderN, VillegasE. Multicountry study of *Aedes aegypti* pupal productivity survey methodology: Findings and recommendations. Dengue Bull., 2007; (31): 192–200. https://iris.who.int/handle/10665/170461.

[25] LounibosLP, EscherRL. Sex ratios of mosquitoes from long-term censuses of Florida tree holes. J. Am. Mosq. Control Assoc., 2008; 24(1): 11–15. doi:10.2987/5656.1.18437808PMC2577382

[26] BlancoK, VillamizarSR, Avila-DiazA Daily dataset of precipitation and temperature in the Department of Cauca, Colombia. Data Brief, 2023; 50: 109542. ISSN 2352-3409, doi:10.1016/j.dib.2023.109542.37743883PMC10514423

[27] GomesH, de JesusAG, QuaresmaJ. Arboviroses of interest in public health transmitted by *Aedes aegypti* in Tocantins: A one health analysis. SSRN Electron. J., 2022; doi:10.2139/ssrn.4210214.

[28] SánchezC, SantamaríaE, MoralesCA Supporting data for “Spatial patterns associated with the distribution of immature stages of *Aedes aegypti* in three high-risk dengue municipalities of Southwestern Colombia”. GigaScience Database, 2023; 10.5524/102439.PMC1062043337928893

[29] Vectors of Human Disease Series. *GigaByte*. 2022; 10.46471/GIGABYTE_SERIES_0002.
